# Metal-Free Fabrication of Fused Silica Extended Nanofluidic Channel to Remove Artifacts in Chemical Analysis

**DOI:** 10.3390/mi12080917

**Published:** 2021-07-31

**Authors:** Kyojiro Morikawa, Ryoichi Ohta, Kazuma Mawatari, Takehiko Kitamori

**Affiliations:** 1 Collaborative Research Organization for Micro and Nano Multifunctional Devices (NMfD), The University of Tokyo, 7-3-1 Hongo, Bunkyo, Tokyo 113-8656, Japan; ohta@icl.t.u-tokyo.ac.jp (R.O.); kitamori@icl.t.u-tokyo.ac.jp (T.K.); 2 Department of Applied Chemistry, School of Engineering, The University of Tokyo, 7-3-1 Hongo, Bunkyo, Tokyo 113-8656, Japan; 3 Institute of Nanoengineering and Microsystems (iNEMS), Department of Power Mechanical Engineering, National Tsing Hua University, No. 101, Section 2, Kuang-Fu Road, Hsinchu 30013, Taiwan

**Keywords:** microfluidics, nanofluidics, extended nanochannel, nanofabrication, fused silica, metal-free

## Abstract

In microfluidics, especially in nanofluidics, nanochannels with functionalized surfaces have recently attracted attention for use as a new tool for the investigation of chemical reaction fields. Molecules handled in the reaction field can reach the single–molecule level due to the small size of the nanochannel. In such surroundings, contamination of the channel surface should be removed at the single–molecule level. In this study, it was assumed that metal materials could contaminate the nanochannels during the fabrication processes; therefore, we aimed to develop metal-free fabrication processes. Fused silica channels 1000 nm-deep were conventionally fabricated using a chromium mask. Instead of chromium, electron beam resists more than 1000 nm thick were used and the lithography conditions were optimized. From the results of optimization, channels with 1000 nm scale width and depth were fabricated on fused silica substrates without the use of a chromium mask. In nanofluidic experiments, an oxidation reaction was observed in a device fabricated by conventional fabrication processes using a chromium mask. It was found that Cr^6+^ remained on the channel surfaces and reacted with chemicals in the liquid phase in the extended nanochannels; this effect occurred at least to the micromolar level. In contrast, the device fabricated with metal-free processes was free of artifacts induced by the presence of chromium. The developed fabrication processes and results of this study will be a significant contribution to the fundamental technologies employed in the fields of microfluidics and nanofluidics.

## 1. Introduction

Miniaturized analytical systems known as microfluidics, micro-total analysis systems (μ-TAS), and lab-on-a-chip have attracted much attention over the past 30 years [1,2]. In this concept, microfluidic channels are fabricated on a substrate and various types of chemical and biochemical processes are integrated into the channels. When compared with bulk processes, there are many advantages with such miniaturized analytical systems, such as ease of analysis, high-speed reaction, low reagent consumption, and small sample volumes. One of the most significant characteristics of these analytical systems is the temporal–spatial uniformity. Considering their typical diffusion time, molecules diffuse over micrometer regions within the order of seconds. Therefore, molecular transport in liquids, even at liquid/liquid interfaces, gas/liquid interfaces, and solid/liquid interfaces, can be completed within a very short time. In addition, considering the small heat capacity of liquids inside a microfluidic channel, the temperature can be instantaneously changed. Such characteristics are essential for the control of chemical reactions, and are not observed in bulk-scale chemical reactions; therefore, microfluidic devices have received much attention as chemical reaction tools. Polymers [3,4,5,6,7,8,9] such as polydimethylsiloxane (PDMS) and polymethyl methacrylate (PMMA), and paper [6,7,10,11,12,13] are widely used as materials for microfluidic devices, mainly for biochemical and bioanalytical devices. The main advantages of these materials include their ease of fabrication and their low cost. On the other hand, glass [9,13,14,15], which has high purity, high chemical and mechanical stability, high optical transparency, and allows facile surface modification, has proven to be a suitable material for chemical and biochemical microfluidic devices. Glass tools such as beakers, pipettes, etc., have been widely used for chemical experiments on the bulk scale, so that there is increasing interest in glass microfluidic devices for chemical experiments in microfluidics.

Since the 2000s, the field of nanofluidics has advanced with downscaling of microfluidics [16,17,18,19,20]. Compared to microspaces, size confinement effects are much more enhanced in nanospaces. A typical volume in a nanospace is from picoliters (pl, 10^−12^ L) to femtoliters (fl, 10^−15^ L), and the number of molecules in the spaces is at the single–molecule level (nM × fl = 1 molecule). In addition, the surface to volume ratio in nanospaces reaches 10^−8^–10^−7^ m^−1^, i.e., molecules in the liquid phase can easily and quickly contact nanochannel surfaces without the use of a mixer. Novel devices for reactions at solid (nanochannel surfaces)/liquid interfaces have been developed using this principle. For example, the biotin–streptavidin reaction [21,22], hybridization of microRNA [23], enzymatic reactions [24], and electrochemical reactions [25,26] have been performed at solid/liquid interfaces in nanochannels. In our group, a picoliter enzymatic reactor [27] using an enzyme-immobilized nanochannel, and femtoliter chromatography devices [28,29] using silica surfaces or C_18_-modified surfaces have been developed, and their superior performance over conventional bulk methods has been verified. A femtoliter enzyme-linked immunosorbent assay (ELISA) [30] using antibody-immobilized nanochannels enabled protein quantification at the single–molecule level. In addition, integration methodologies known as micro unit operations (MUOs), nano unit operations (NUOs), and continuous flow chemical processing (CFCP) [31] have been used to realize devices for living single–cell analysis [32]. Therefore, nanochannels with functionalized surfaces can act as a reaction field, and thus nanofluidics has received much attention as a chemical reaction tool.

However, a countermeasure to contamination is essential in the use of nanochannels, and this should be performed at the single–molecule level because molecules in nanochannels are handled at this level. Contamination during nanochannel fabrication processes is no exception. Electron beam (EB) lithography, photolithography, and dry etching are the main processes we employ for the fabrication of fused silica nanochannels/microchannels [33]. In these processes, different etching masks are used based on the target channel dimensions. To select the etching masks, mainly two factors should be considered: lithography resolution and etching resistivity. For example, an EB resist can be used to perform lithography with 100 nm scale resolution, but channel depth will be less than 100 nm scale after etching due to etching selectivity of the EB resist/fused silica. A photoresist can be used to perform lithography with 1 μm scale resolution, but channel depth will be less than 1 μm scale after etching due to etching selectivity of the photoresist/fused silica. To improve etching resistance while keeping the lithography resolution, a combination of EB resist and metal mask or combination of photoresist and metal mask are widely used. In our group, combination of EB resist and metal mask (mainly chromium) are used to fabricate channels where the width and depth are 500–5000 nm (which is called extended nanochannels [19]), and combination of photoresist [34] and chromium are mainly used to fabricate microchannels where the width and depth are 5–50 μm. Chromium contributes very well to these fabrication process, as well as conventional fabrication methods. However, if chromium or its oxidation products remain on extended nanochannel surfaces, they have a negative effect on chemical reactions in the extended nanochannel because they are active for redox reactions. The reaction derived from chromium can disturb target chemical reaction in the extended nanochannel; artifacts from chromium reaction can be induced. For example, TMB (3,3′,5,5′-tetramethylbenzidine) is widely used as a substrate in enzymatic amplification reaction for protein analyses such as ELISA, Western blotting, etc. Redox reactions between chromium and TMB can disturb target enzymatic reaction of TMB; as a result, detection of signals from a target enzymatic reaction will be difficult. Therefore, chromium-free fabrication methods are desirable to avoid this problem. A photoresist can be used instead of chromium masks for microchannel fabrication; however, it is difficult to find an alternative to chromium for the fabrication of extended nanochannels. One typical approach to the fabrication of extended nanochannels without chromium is the use of a thicker EB resist (the thickness of a conventional resist is less than 1000 nm [35]). However, considering the lithography principle, the formation of narrower lines is difficult with a thicker resist. There is little information on the use of thick resists because the research trend is mainly aimed at the fabrication of narrower nanochannels using thinner resists, as typified by the fabrication of semiconductor silicon [36,37]. In addition, fused silica has higher etching resistivity than silicon; therefore, a thicker resist would be required for the fabrication of fused silica extended nanochannels, which indicates more difficult fabrication.

In this study, a process for the fabrication of fused silica channels with widths and depths on the scale of 1000 nm is presented. To realize this process, an EB resist with a thickness of approximately 2000 nm was coated on fused silica. The EB exposure time was optimized for the thick resist, and the channel dimensions after etching were evaluated. Finally, artifact removal from analyses was evaluated. As a model sample, TMB was used, and devices fabricated by conventional chromium mask processes and those fabricated by the metal-free process developed were used in this study.

## 2. Materials and Methods

**Figure 1:**
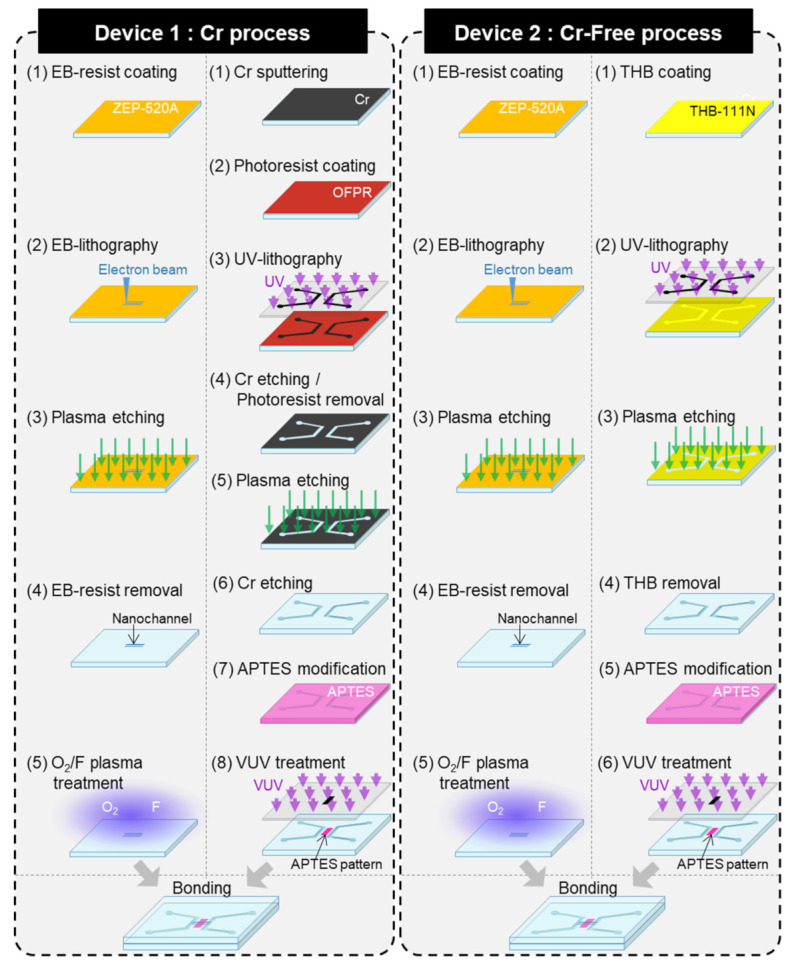
Processes for fabrication of nanofluidic devices using fused silica substrates.

Figure 1 shows the various fabrication processes for the nanofluidic devices. First, an EB resist (ZEP-520A, Zeon Corp., Tokyo, Japan) was spin-coated onto a fused silica substrate (VIO-SILSX, Shin-Etsu Quartz Co., Ltd., Tokyo, Japan; 70 × 30 × 0.7 mm). After spin-coating, the substrate was heated at 180 °C for 2 min. The substrate was then spin-coated again using the same spin speed. After the second spin-coating, the substrate was again heated at 180 °C for 2 min. In these processes, three types of substrates were prepared with different spin-coating speeds of 500, 700, and 1000 r/min. EB lithography was subsequently performed. The exposure area was designed as lines and spaces, where the width of the lines was 1000, 1500, 2000, 2500, 3000, 3500, and 4000 nm with various exposure times (0.2, 0.3, 0.4, 0.5, 0.6, 0.7, 0.8, 0.9, or 1.0 μs/dot; 1 dot = 5 × 5 nm area). After EB lithography, development was performed by dipping in *o*-xylene, and the thickness of the EB resist was then measured. Dry etching was then performed using an NLD-570 system (Ulvac Co., Ltd., Kanagawa, Japan) with gaseous SF_6_ and CHF_3_. The etching time for the substrate coated twice at 500 r/min was 1200 s, that coated twice at 700 r/min was 1000 s, and that coated twice at 1000 r/min was 800 s. The etching profiles of the channels were measured with a stylus profiler (dektakXT-A, Bruker Corp., Billerica, MA, USA) and an optical profiler (WYKO NT9100A, Bruker Corp., Billerica, MA, USA).

Microchannels for liquid introduction with a width of 500 μm and a depth of 2 μm were fabricated on another substrate. Two different devices were prepared for verification of artifact removal. The processes for fabrication of these two devices were based on fl-ELISA devices [30]. In device 1, microchannels were fabricated using a chromium mask and a photoresist material (OFPR-800, Tokyo Ohka Kogyo Co., Ltd., Tokyo, Japan). In these processes, photolithography was performed on the substrate coated OFPR-800 photoresist and chromium. After the development of an OFPR-800 photoresist with dipping in tetramethylammonium hydroxide (TMAH) solution, chromium was etched by dipping in a chromium etchant solution (Ce(NH_4_)_2_(NO_3_)_6_). In device 2, THB type (no metal) photoresist (JSR Corp. Tokyo, Japan) [38] was used to fabricate microchannels for comparison with the effects of chromium. Photolithography was performed on the substrate coated THB type photoresist, and after that, development was performed by dipping in TMAH solution. The difference between devices 1 and 2 was the different microchannel fabrication process with/without chromium. After that process, the substrates of both devices 1 and 2 were patterned with amino-propyltriethoxysilane (APTES). Each type of substrate with microchannels was bonded to another substrate with extended nanochannels by a low-temperature bonding method [39]. The extended nanochannels for devices 1 and 2 were fabricated by the following method: twice coating at 1000 r/min, 2000 nm exposure area, 0.6 μs/dot exposure time, and dry etching with 720 s. Prior to this bonding process, fluorine was added during plasma treatment of the extended nanochannel fabricated substrates. Vacuum ultraviolet (VUV) treatment was performed for the microchannel fabricated substrates. After bonding, the channel surfaces were modified with polyethylene glycol (PEG).

**Figure 2:**
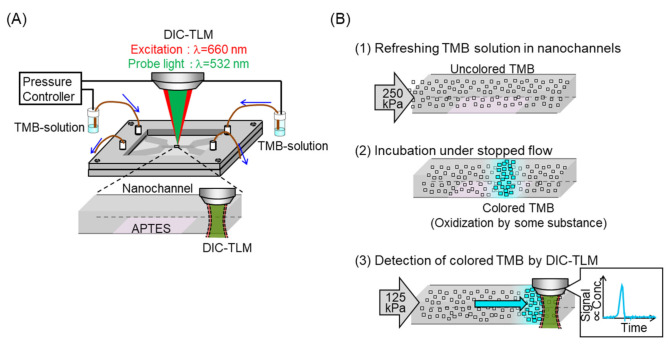
Evaluation of artifact on nanofluidic ELISA. (**A**) Setup. (**B**) Procedure of fluidic control.

The obtained devices were used to evaluate the artifacts in chemical analysis. The setup is shown in Figure 2A. SureBlue TMB 1-Component (KPL, Gaithersburg, MD, USA), aqueous solution including 1.7 mM TMB (3,3′,5,5′-tetramethylbenzidine), was introduced into extended nanochannels via microchannels and capillaries by pressure controllers. The fluidic control procedure is shown in Figure 2B). This operation was modeled after nanofluidic ELISA [30]. At first, TMB solution in the extended nanochannel was refreshed by applying 250 kPa pressure. Then, flow was stopped at 0.5, 1, 2, and 4 min for the incubation. At this time, uncolored TMB might be colored if oxidatively active contamination was present. After that, flow was started again. Signals associated with oxidized TMB (absorption max. wavelength at 655 nm [40]) were detected using a differential interference contrast thermal lens microscope (DIC-TLM; excitation: 660 nm, probe: 532 nm) [41]. The shape of the signals was expected to reflect the concentration distribution of oxidized TMB.

## 3. Results and Discussion

**Figure 3:**
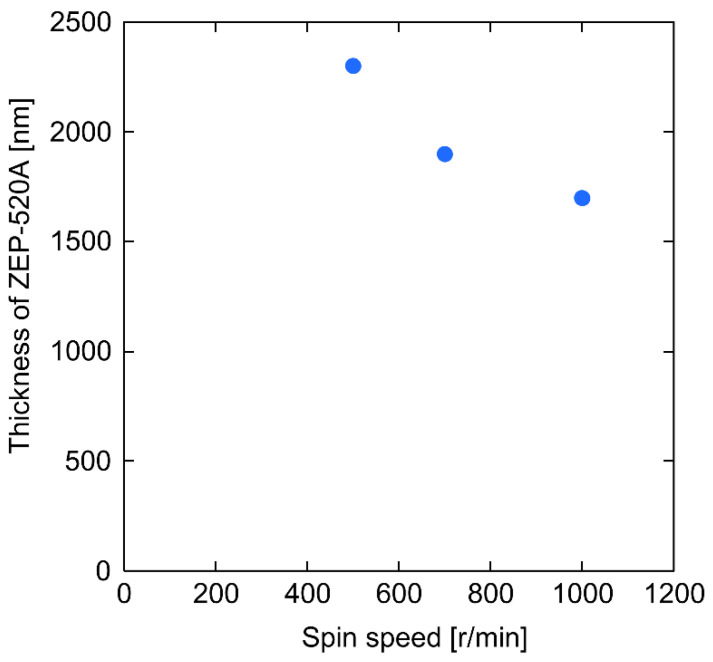
Thickness of spin-coated ZEP-520A with double coating.

**Figure 4:**
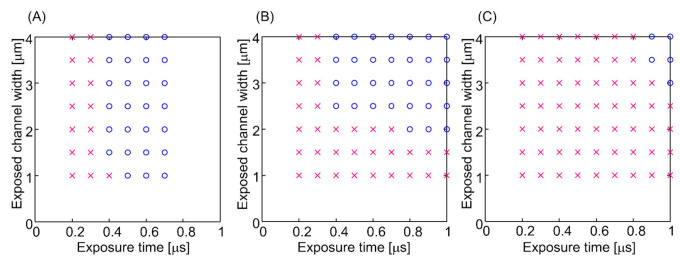
Relationship between exposure time and exposed channel width. Circles indicate conditions for successful channel fabrication, cross marks indicate conditions for failed channel fabrication. ZEP-520A EB resist with thicknesses of (**A**) 1700 nm, (**B**) 1900 nm, and (**C**) 2300 nm.

**Figure 5:**
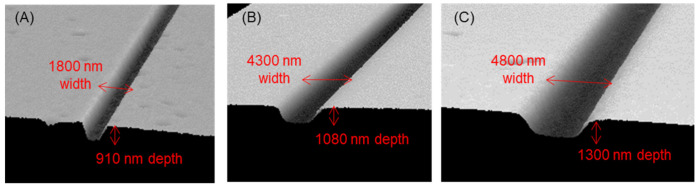
3D profiles of fabricated extended nanochannels scanned by an optical profiler (WYKO NT9100A). Results for fabrication with (**A**) 1700 nm resist thickness, 1000 nm exposed channel width, and 0.5 μs exposure; (**B**) 1900 nm resist thickness, 2500 nm exposed channel width, and 0.4 μs exposure; and (**C**) 2300 nm resist thickness, 3000 nm exposed channel width, and 1.0 μs exposure.

Figure 3 show the results for the thickness of ZEP-520A after spin-coating twice. A thicker resist was obtained with a lower spin speed, with the maximum thickness being more than 2000 nm. The etching selectivity of (fused silica)/(ZEP-520A) was approximately 0.5; therefore, channels deeper than 1000 nm could be produced by etching. The results of EB lithography and dry etching are shown in Figure 4. For the 1700 nm-thick resist in Figure 4A, almost entirely 1000 nm scale channels could be fabricated for a >0.4 μs exposure. For the 1900 nm-thick resist in Figure 4B, channels wider than 2500 nm could be fabricated with >0.4 μs exposure, although an exposure time of >0.8 μs was required for 2000 nm-wide channels. No 1000–1500 nm channels were fabricated under these conditions. For the 2300 nm-thick resist in Figure 4C, a longer exposure time (0.9–1.0 μs) and wider exposure area (3000–4000 nm width) were required for channel fabrication. These results are quite reasonable because a longer exposure time and wider exposure area are required to complete lithography in the depth direction with thicker resists. These are the first reported lithography conditions for EB resists more than 1000 nm thick. After etching, a depth of 910 nm was obtained with the 1700 nm-thick resist, a 1080 nm depth with the 1900 nm-thick resist, and a 1300 nm depth with the 2300 nm-thick resist. Typical results are shown in Figure 5. Figure 5A shows that a channel with a width of 1800 nm and a depth of 910 nm was successfully fabricated, and channels wider than 1000 nm could be fabricated based on the lithography principle (easier formation of wider channels). In addition, a channel with a width of 4300 nm and a depth of 1080 nm, and a channel with a width of 4800 nm and a depth of 1300 nm, were successfully fabricated, as shown in Figure 5B,C, and channels wider than 3000 nm could be fabricated under this channel depth condition. Fabrication of extended nanochannels whose width and depth are 1000 nm scale on a fused silica substrate is difficult by conventional methods [42]. For example, using mechanical methods such as blasting and milling, it is difficult to fabricate 1000 nm scale channels due to their resolution. In addition, optical methods and electrical methods are difficult to use to fabricate size- and shape-regulated extended nanochannels. Using the basic methods by lithography and etching, metal masks are necessary to fabricate 1000 nm scale channels. Therefore, 1000 nm scale channels were successfully fabricated on fused silica substrates without the use of chromium masks for the first time.

**Figure 6:**
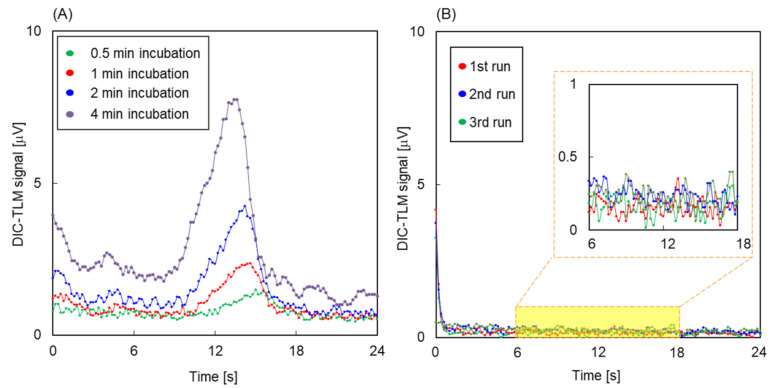
(**A**) DIC-TLM signals during 24 s flow with different incubation times for device 1. (**B**) DIC-TLM signals during 24 s flow in the 3 times experiments.

**Figure 7:**
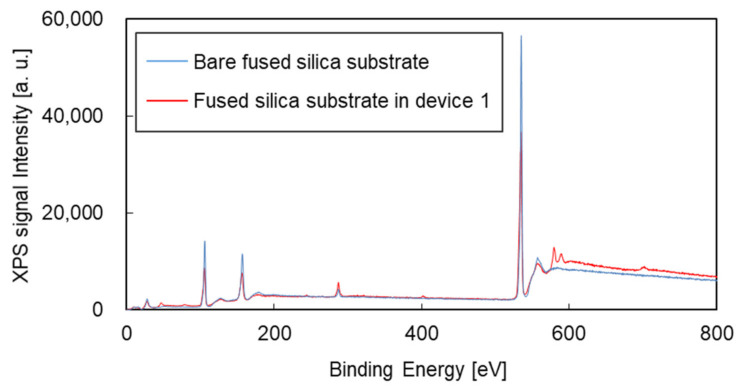
XPS signals for fused silica substrates before and after channel fabrication.

Figure 6 shows DIC-TLM signals for TMB solutions in devices 1 and 2. The sizes of extended nanochannels of devices 1 and 2 were 2400 nm width and 800 nm depth, which showed successful fabrication by 1700 nm-thick resist with two coatings at 1000 r/min in Figure 3, and by exposure of 2000 nm area with 0.6 μs/dot in Figure 4A. As shown in Figure 6A, signals were obtained that indicated the oxidation of TMB, and the progress of the reaction is indicated by the increase in the signal intensity with increasing incubation time. ZEP-520A, OFPR, APTES and PEG (and of course SiO_2_) do not readily react with TMB. Therefore, chromium is the only candidate for reaction with TMB. Figure 6B shows the DIC-TLM signals for TMB solutions in device 2. Device 2, which was fabricated using metal-free processes, was free from artifacts. The results presented here indicate that the presence of chromium in channels can induce artifacts, whereas such artifacts can be removed through the use of metal-free fabrication processes. In previous reports on ELISA in microchannels fabricated using a chromium mask on fused silica substrates [43,44,45], the artifacts induced by chromium were not discussed. It was suggested that effects of remnants of chromium on the microchannel surface were not significant due to lower surface-to-volume ratio compared to that of the extended nanochannel. Therefore, the artifacts induced by remnants of chromium are considered to be significant only in extended nanochannels. However, without metal-free fabrication processes developed in this study, the artifacts induced by remnants of chromium were difficult to verify. Therefore, this is the first finding that chromium remaining on the extended nanochannel surfaces during fabrication induced artifacts during the analysis. X-ray photoelectron spectroscopy (XPS) measurements were performed to evaluate the material composition on the fabricated substrates, and the results are shown in Figure 7. Around 580 eV, clear peaks were found for the substrate of device 1, but these were absent for a bare fused silica substrate. Based on the NIST XPS database [46], Cr 2p in CrO_3_ is observed at 579 eV; therefore, the results indicated that Cr^6+^ remained on the channel surfaces, reacted with THB, and induced artifacts.

The amount of reacted Cr^6+^ was estimated to elucidate the Cr^6+^ effect. Calibration of the DIC-TLM signals indicated that the reaction rate of TMB in the extended nanochannel was 3.6 μM/min. For comparison, the TMB reaction rate was measured using a 20 μM K_2_CrO_7_ solution, for which the rate was 0.33 μM/min. We have previously reported that the reaction rate in nanochannels can be increased by 10^1^ times due to size confinement effects [27]. Therefore, whether or not to consider the size confinement effects would require at least a micromolar order (apparent concentration by nanochannel surface-to-volume ratio) reaction of Cr^6+^, which would have a significant effect on the chemical process. This is the first finding that chromium remaining on the channel surfaces during fabrication induced artifacts during the analysis, and the effect was significant. This finding will be valuable in designing nanofluidic devices and experiments.

## 4. Conclusions

In this study, we presented processes for the fabrication of 1000 nm scale channels on fused silica substrates without using a metal mask. With control of the spin-coating speed, an EB resist was coated on the substrates with thicknesses in the range of 1700–2300 nm. The EB exposure conditions for such thick resists were then optimized. As a result, channels with 1000 nm scale widths and depths were fabricated on fused silica substrates without the need for a metal mask. This is the first fabrication of 1000 nm scale channels without using a metal mask. DIC-TLM signals due to oxidized TMB were obtained for a nanofluidic device fabricated using a chromium mask. Chromium remaining on the channel surfaces during fabrication induced artifacts during the analysis, and the effect was significant at micromolar levels. This is the first finding that chromium remaining on the channel surfaces during fabrication induced artifacts during the analysis. The results of this study are expected to be valuable for the design of nanofluidic devices and experiments. In addition, metal-free micro/nanochannel fabrication processes can be realized to remove artifacts due to metal contamination, which will serve to advance the fundamental technologies employed in microfluidics and nanofluidics.
